# Distinctive effects of fear and sadness induction on anger and aggressive behavior

**DOI:** 10.3389/fpsyg.2015.00725

**Published:** 2015-06-15

**Authors:** Jun Zhan, Jun Ren, Jin Fan, Jing Luo

**Affiliations:** ^1^Beijing Key Laboratory of Learning and Cognition, The Collaborative Innovation Center for Capital Education Development, Department of Psychology, Capital Normal University, Beijing, China; ^2^Department of Psychology, Zhejiang Normal University, Jinhua, China; ^3^Department of Psychology, Queens College, City University of New York, New York, NY, USA; ^4^Key Laboratory of Mental Health, Institute of Psychology, Chinese Academy of Sciences, Beijing, China

**Keywords:** anger, sadness, fear, aggressive behavior, emotion regulation, mood induction

## Abstract

A recent study has reported that the successful implementation of cognitive regulation of emotion depends on higher-level cognitive functions, such as top-down control, which may be impaired in stressful situations. This calls for “cognition free” self-regulatory strategies that do not require top-down control. In contrast to the cognitive regulation of emotion that emphasizes the role of cognition, traditional Chinese philosophy and medicine views the relationship among different types of emotions as promoting or counteracting each other without the involvement of cognition, which provides an insightful perspective for developing “cognition free” regulatory strategies. In this study, we examined two hypotheses regarding the modulation of anger and aggressive behavior: sadness counteracts anger and aggressive behavior, whereas fear promotes anger and aggressive behavior. Participants were first provoked by reading extremely negative feedback on their viewpoints (Study 1) and by watching anger-inducing movie clips (Study 2). Then, these angry participants were assigned to three equivalent groups and viewed sad, fear-inducing, or neutral materials to evoke the corresponding emotions. The results showed that participants displayed a lower level of aggressive behavior when sadness was later induced and a higher level of anger when fear was later induced. These results provide evidence that supports the hypothesis of mutual promotion and counteraction relationships among these types of emotions and imply a “cognition free” approach to regulating anger and aggressive behavior.

## Introduction

Psychological studies have illustrated that there are many different approaches to the exertion of self-regulatory control over unwanted feelings, thoughts, and behavior (Ochsner and Gross, 2005; Webb et al., 2012), including attention control (e.g., selective attention and distraction; Bantick et al., 2002; Tracey, 2002; Kanske et al., 2010) and cognitive change (e.g., reappraisal; Ochsner et al., 2002; Ochsner and Gross, 2008). When self-regulatory control is implemented, one must typically utilize his/her cognitive ability (Todd et al., 2010). As the regulatory strategy becomes more deliberate, a more precise and complicated cognitive process is needed (Todd et al., 2012). However, the efficiency of this cognition-based regulation was challenged by a recent study that found a “prefrontal cortex (PFC) function paradox” in cognitive regulation (Raio et al., 2013). That is, cognitive emotion regulation relies on higher or at least normal cognitive function of the PFC, but this function can be impaired by stress (Arnsten, 2009). As a consequence, the implementation of cognitive regulation in a stressful situation is disrupted. This paradox calls for different regulation strategies, in which efficiency may not heavily depend on higher-level cognitive regulation and PFC function.

**FIGURE 1:**
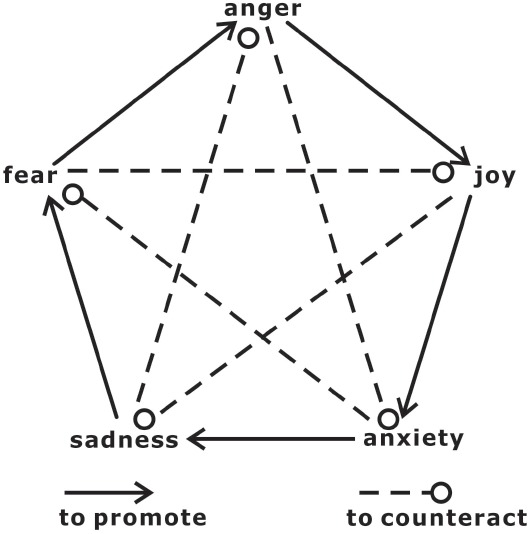
**The relationships between mutual promotion and mutual restraint and the emotions of joy, thinking/anxiety, sadness, fear, and anger.** The promotion relationships include the following: joy promotes thinking/anxiety, thinking/anxiety promotes sadness, sadness promotes fear, fear promotes anger, and anger promotes joy. The restraint relationships include the following: joy counteracts sadness, sadness counteracts anger, anger counteracts thinking/anxiety, thinking/anxiety counteracts fear, and fear counteracts joy.

In this study, we introduced a novel “cognition-free” regulatory strategy based on theories of traditional Chinese philosophy and medicine. In contrast to cognitive emotion regulation, which emphasizes the role of cognition in the implementation of top-down regulation of emotion (Ochsner et al., 2002, 2012), traditional Chinese philosophy and medicine views different types of mental states and emotions as having mutual promotion and counteraction (allelopathy) relationships. More specifically, mutual promotion and mutual restraint exist among the emotions of anger, joy, thinking/anxiety, sadness, and fear. The promotion relationship is expressed in the following manner: joy promotes thinking/anxiety, thinking/anxiety promotes sadness, sadness promotes fear, fear promotes anger, and anger promotes joy. The restraint relationship is expressed as follows: joy counteracts sadness, sadness counteracts anger, anger counteracts thinking/anxiety, thinking/anxiety counteracts fear, and fear counteracts joy (Figure 1). The mutual promotion and restraint model of different types of emotions involves many specific hypotheses, and it will be difficult, if not impossible, to scientifically consider and test all of these hypotheses in a single study. However, some of these hypotheses are reasonable and testable even from the view of modern emotion science. More importantly, they may provide an original perspective of emotion regulation that is essentially different from the mechanism of cognitive top-down control.

The aim of the present study was to experimentally examine two hypotheses of the above-mentioned model on the modulation of anger: sadness counteracts (or alleviates) anger and fear promotes (or reinforces) anger. Thus, inducing the emotion of sadness or fear may alleviate or promote the already evoked emotion of anger. Take the hypothesis of “sadness counteracts anger” as an example: classic traditional Chinese medicine recorded cases of patients who had illnesses that were caused by anger and could be cured through inducing the emotion of sadness (see Jiang and Wei, 1996, for a review). From the perspective of emotion science, the mechanisms of “sadness counteracts (or alleviates) anger” and “fear promotes (or reinforces) anger” can be understood by examining the interaction between anger and fear or between anger and sadness. Fear is similar to anger in that it involves the processing of signals of threat to the individual (Cannon, 1929). A study that adopted the terminology of Buckner et al. (2011) for networks found that the anger and fear categories were both characterized by a profile that mainly involved the “dorsal attention,” “visual” (occipital), “frontoparietal,” “limbic,” and “default mode” networks (Wager et al., 2015). In contrast, the emotion of sadness originates from the child’s separation from an early symbiosis with the mother and was found to be similar to happiness in terms of the opioid-based mechanisms (Panksepp, 1998) and the cortical patterns of activation. Specifically, patterns for both the sadness and happiness categories were characterized by moderate activation of the profile that characterized anger and high intensity in the profile that included the “ventral attention,” “somatomotor,” and “visual” networks (Wager et al., 2015). Therefore, the induction of fear over the already evoked anger emotion may increase one’s anger and/or aggressive feelings, whereas the induction of sadness may do the reverse.

In this study, we first provoked all participants to become angry. Then, we divided these participants into three groups that watched different emotion-inducing movie clips to induce sadness, fear, or neutral emotion to examine the effects of mood induction (MI) of these emotions on participants’ anger and aggressive behavior. This “anger first, MI later” procedure was utilized based on previous experimental designs that tested the catharsis hypothesis of anger modulation (e.g., reducing anger and aggressive feelings by hitting a punching bag; Bushman et al., 1999). It also largely simulated the situation of the “sadness counteracts anger” treatment recorded in the classic works of traditional Chinese medicine (e.g., Jiang and Wei, 1996).

One point that should be noted in this study is that the experimental procedure did not test whether inducing the emotion of sadness or fear in a peaceful (or an emotionally neutral) mind could reduce or increase one’s “absolute” level of angry feelings; rather, it tested how inducing the emotion of sadness or fear could alter (alleviate or promote) the progress of the already evoked emotion of anger. Specifically, for the hypothesis of “fear promotes anger,” we could make two types of predictions. One prediction is that the anger level of the already provoked individual is even higher after inducing fear; this possibility is true only when the promoting effect of fear on anger is sufficiently robust to override the natural decline of the experimentally evoked anger. A second possibility is that the induction of fear cannot cause the provoked individual to become angrier than he/she was before this induction but can slow down the individual’s recovery from the first anger induction (AI). This prediction, although is less robust than the first, can also confirm the hypothesis of “fear promotes anger” as long as the induction of fear slows the recovery from the first AI relative to the induction of other types of emotions, such as neutral or sad emotions.

An additional point that should be noted is that both participants’ subjective feelings of anger and their aggressive behavior were evaluated in this study. Although different from anger, aggressive behavior is often positively correlated with angry feelings (Bushman et al., 1999; Parrott and Giancola, 2007) and is presented as a consequence of anger (Bandura et al., 1963; Novaco, 1994; Lieberman et al., 1999).

## Study 1

### Methods

#### Participants

A total of 93 undergraduate and graduate students from universities in Beijing participated in this experiment. The data from three participants were excluded from the final analysis because two participants failed to experience anger and one participant had correctly guessed the purpose of the experiment before it started. The remaining 90 participants, who ranged in age from 20 to 25 years, with a mean of 22 years, were randomly subdivided into the fear, sadness, and neutral emotion groups, with each group consisting of 30 participants (with equal numbers of males and females). All of the participants signed the informed consent form for the experiment, and each participant was compensated 25–30 RMB for participating in the study.

#### Experimental Design and Procedures

**FIGURE 2:**
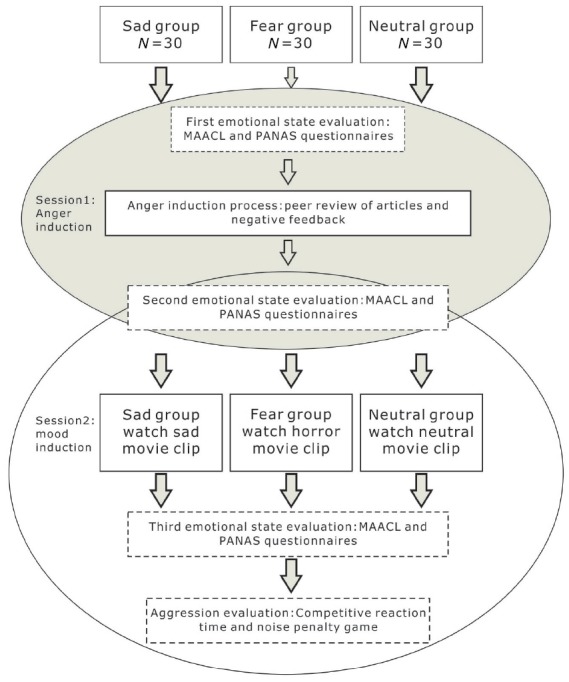
**Schematic illustration of the experimental procedure in Study 1.** The procedure of Study 2 was the same except that participants watched anger-inducing movie clips in the anger induction stage.

The entire experimental procedure consisted of two stages (Figure 2). First, anger was induced in all three groups (AI session). Subsequently, sadness, fear, and neutral emotion inductions were conducted separately for each group to examine the regulatory effect of these emotions on anger and aggressive behavior at the second stage (MI session). There were also three emotional evaluations: the first emotional evaluation was administered before AI, the second was administered after AI, and the third was administered after sadness/fear/neutral emotion induction. The aim of these evaluations was to estimate the participants’ immediate feelings of anger and positive and negative emotions at each stage. Finally, aggressive behavior was assessed using a competitive computer game (Taylor, 1967).

Participants were individually tested, and each participant was led to believe that he or she would be interacting with another participant. When a participant arrived at the laboratory, the experimenter first asked the participant to complete the questionnaires regarding his/her feelings of anger and positive and negative emotions at that time. Feelings of anger were measured using the hostility subscale of the revised Multiple Affect Adjective Checklist (MAACL; Zuckerman and Lubin, 1985) according to the method reported in a related experiment performed by Bushman et al. (2001). In the Chinese version of the MAACL (Zhang, 1991), the hostility subscale contains 22 adjectives, including 11 words that are positively associated with anger (irritable, cruel, jealous, disgruntled, indignant, impatient, hostile, irritated, violent, furious, and exasperated) and 11 words that are negatively associated with anger (gracious, easy-going, good-natured, helpful, friendly, courteous, gentle, pleasantly agreeable, kind, affable, and cooperative). The participants were asked to check these 22 adjectives according to their feelings at that time. When they selected a word that was positively associated with anger or unselected a word that was negatively associated with anger, they accumulated one point; the final scores were the sum of the total points. High total scores indicate a high level of anger. We also used the Positive and Negative Affect Schedule (PANAS; Watson and Tellegen, 1985) to assess the participants’ emotional states. This scale was used in a similar study (Bushman et al., 2001) and the applicability of the Chinese version of the PANAS scale was confirmed in previous studies (Huang et al., 2003). Positive affect was measured using 10 adjectives from the positive affect subscale of the PANAS; negative affect was measured using 10 adjectives from the negative affect subscale of the PANAS. All of the adjectives were rated along a 5-point Likert-type scale: 1 (very slightly or not at all), 2 (a little), 3 (moderately), 4 (quite a bit), and 5 (extremely). The participants were told to “indicate the extent to which you feel this way right now, that is, at the present moment.” The scores for the adjectives of the positive affect subscale and negative affect subscale were added to obtain the levels of positive and negative emotions, respectively.

The AI procedure that was utilized in previous studies (Bushman et al., 2001; Bushman, 2002) was administered after the participants completed the questionnaires. The participants were asked to write a paragraph that focused on a popular topic in Chinese societies (e.g., the new marriage law on property division in divorce) and to express their views on the subject. The experimenter told each participant that another participant was completing the questionnaire in another room (this participant did not actually exist), that they would later evaluate each other’s views using a score ranging from –10 (very poor) to 10 (very good) and that they would provide a brief comment. When the participant completed the writing of his/her viewpoint, the experimenter took the comments and claimed that the comments would be reviewed by the other participant. The experimenter also presented the “other” participant’s paper (in fact, the experimenter had prepared it in advance) to the participant and asked him/her to carefully read the paper, assign a score, and make a brief comment. Subsequently, the experimenter showed the participant the extremely negative evaluation of his/her viewpoint, which was made by the “other” participant. Previous studies have shown that this procedure makes people angry and increases their aggressive behavior (Bushman and Baumeister, 1998; Bushman et al., 1999, 2001; Bushman, 2002). After reading the evaluation, the participant was asked to complete the hostility subscale of the MAACL and the PANAS questionnaires.

Then, participants were randomly assigned to one of three groups to watch different emotional movie clips to induce sadness, fear, or neutral emotions. This method has the advantages of simplicity and authenticity with audiovisual dual-channel stimulus input (Gross and Levenson, 1995; Gross, 1998; Frazier et al., 2004). The movie clips were extracted from the Chinese Emotional Visual Stimulus (CEVS) database (Xu et al., 2010). This study used three video clips for inducing sadness (duration, 2 min 16 s; from the movie “Mom Love Me Once Again”; intensity, *M* = 3.17, SD = 1.56), fear (duration, 2 min 17 s; from the movie “Help”; intensity, *M* = 3.33, SD = 2.1), and neutral emotion (duration, 2 min 17 s; from the movie “Computer Repair”; intensity, *M* = 1.0625, SD = 0.25). While watching the movie, participants were asked to attempt to be as attentive to the movie as possible and to express their natural feelings and not suppress any emotion. Finally, participants were requested to complete the hostility subscale of the MAACL and the PANAS questionnaires.

Subsequently, the Taylor Aggression Paradigm (TAP, Taylor, 1967) was used to measure the participants’ aggressive behavior. Each participant was informed that he/she would be paired with another player, without meeting in person, to play a competitive game. The other player was the person who provided negative reviews regarding the arguments made by the participant; thus, it was likely and natural for participants to vent their anger and show aggressive behavior (Bushman, 2002). In the game, the participant was asked to respond as quickly as possible by pushing the button after the appearance of the signal. In each round, the player with a slower reaction time was the loser and would receive noise punishment by the winner (i.e., the participant who responded quicker). The reaction time task consisted of 25 trials. Before the start of each round of the competitive game, the participant had to pre-set the decibel level of the noise that the opponent would be subjected to. The so-called companion did not exist, and all decibel levels were preset by the experimenter. The study design controlled the noise intensity in the range of 60 (Level 1) to 105 decibels (Level 10), which was selected by the participants (DeWall et al., 2007). All of the noises were produced using the Praat speech software (a free scientific computer software package that was designed by Paul Boersma and David Weenink of the University of Amsterdam). Each noise had a 2-s duration, and the average noise intensity set by the participant in 25 rounds was used to indicate the participant’s aggressive behavior, which was believed to be associated with the previous AI procedure. The construct validity of this paradigm has been verified by previous studies (Bernstein et al., 1987; Giancola and Zeichner, 1995), including a study that was similar to the present study (Bushman, 2002).

After the experiment was completed, the experimenter thanked the participants for participating in the trial and compensated them for their participation. The experimenter also asked the participants to talk about their understanding of the experimental procedures, the feelings that they experienced after watching the movies, and whether they could guess the purpose of the experiment. After completing all of the experimental procedures and inquiries, the experimenter immediately informed the participants about all aspects of the experiment to minimize the potential adverse psychological impact on the participants caused by the AI program. The experimenter also asked the participants not to discuss the experimental procedures with others to prevent future participants from acquiring knowledge of this experiment. Participants who guessed the purpose of the experiment, who were not angered, or who were not induced to experience the emotion of sadness or fear were excluded from the analysis.

#### Data Analysis

To examine the effects of the two emotion induction procedures on participants’ subjective feelings of anger and their positive and negative emotions, we performed two repeated measures ANOVAs on these emotional scores. One ANOVA was conducted to examine the effects of the AI procedure; the other was conducted to examine the effects of the fear/sadness/neutral emotion induction procedure. These ANOVAs were conducted as 2 (time: first emotional evaluation, second emotional evaluation) × 3 (group: fear group, sad group, neutral emotion group) and 2 (time: second emotional evaluation, third emotional evaluation) × 3 (group: fear group, sad group, neutral emotion group) mixed designs. Moreover, a one-way ANOVA on the competitive game scores was performed to investigate aggressive behavior under three different experimental conditions. All of the dependent variables were subjected to homogeneity of variance tests prior to the main analyses. This test indicated that ANOVA was an acceptable method of handling these data. Given that ANOVAs with balanced designs are robust to moderate deviations from normality, the normality assumption underlying ANOVA is less important, especially when the samples are sufficiently large, unless there is evidence of the non-normality of the population (Bartlett, 1935; Daniels, 1938; Box and Andersen, 1955; Boneau, 1960; Casella and Berger, 2002; Schmider et al., 2010). Therefore, the reliability of our statistical inference is not likely to be influenced by the results of the normality check, as the sample size of each group in our studies (Study 1 and Study 2) reached 30 (Pearson, 1931; Gayen, 1950; David and Johnson, 1951).

### Results

#### Effects of Anger Induction

***Subjective feelings of anger***

**FIGURE 3:**
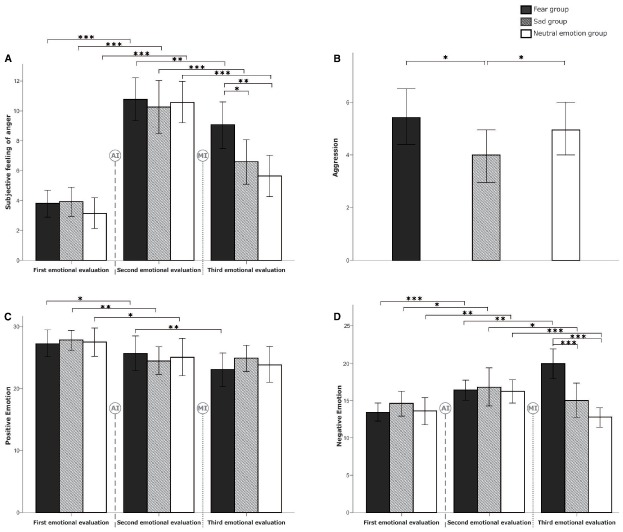
**Comparisons of emotional change and the level of aggressive behavior among the three experimental conditions in Study 1.** Participants’ subjective feelings of anger, positive emotion, and negative emotion in the three experimental stages (i.e., first emotional evaluation, which was administered before anger induction; second emotional evaluation, which was administered after anger induction; and third emotional evaluation, which was administered after the fear/sadness/neutral emotion induction) are demonstrated in **(A,C,D)**, respectively. The levels of aggressive behavior, which were tested after the fear/sadness/neutral emotion induction, are presented in **(B)**. The signs of AI and MI, indicated with a dashed line, represent the anger induction and mood induction manipulations, respectively. The error bars (capped vertical bars) represent 95% confidence intervals. Significant difference at *p < 0.05, **p < 0.01, ***p < 0.001.

The repeated measures ANOVA on feelings of anger showed that the main effect of time was significant [*F*_(1,29)_ = 214.391, *p* = 0.000, η^2^ = 0.711], indicating that the mean level of anger after AI was significantly higher than that before AI. This result confirmed the effectiveness of AI (Figure 3A). However, neither the main effect of group (*F* < 1, η^2^ = 0.004) nor the interaction between time and group (*F* < 1, η^2^ = 0.010) was significant.

***Positive emotion***

The repeated measures ANOVA of positive emotion, as measured by the PANAS positive affect subscale, showed that the main effect of time was significant [*F*_(1,29)_ = 20.951, *p* = 0.000, η^2^ = 0.194; Figure 3C], revealing that the positive emotion level after AI was significantly lower than that before AI. However, neither the main effect of group (*F* < 1, η^2^ = 0.001) nor the interaction between time and group (*F* < 1, η^2^ = 0.02) was significant.

***Negative emotion***

The repeated measures ANOVA of negative emotion, as measured by the PANAS negative affect subscale, showed that the main effect of time was significant [*F*_(1,29)_ = 26.944, *p* = 0.000, η^2^ = 0.236], revealing that the negative emotion after AI was significantly increased compared to the level before AI (Figure 3D). However, neither the main effect of group (*F* < 1, η^2^ = 0.009) nor the interaction between time and group (*F* < 1, η^2^ = 0.004) was significant.

In summary, the comparison before and after the procedure of AI and the comparison among groups indicated the following: (a) the AI procedure was efficient and significantly increased the level of anger and negative emotion and decreased the level of positive emotion and (b) there was no group difference in feelings of anger, positive emotion, or negative emotion before or after the AI procedure. These results provided a suitable basis for conducting the MI manipulations.

#### Effects of Fear and Sadness Induction on Anger and Aggressive Behavior

***Feelings of anger***

The repeated measures ANOVA of anger showed that the main effect of time was significant [*F*_(1,29)_ = 47.727, *p* = 0.000, η^2^ = 0.354]. The anger level after these fear/sadness/neutral emotion inductions was significantly lower than that before the emotion inductions. The main effect of group was marginally significant [*F*_(2,87)_ = 2.609, *p* = 0.079, η^2^ = 0.057]. The interaction between time and group was significant [*F*_(2,87)_ = 3.482, *p* = 0.035, η^2^ = 0.074]. A simple comparison that focused on the group differences showed that before induction, there was no significant group difference (*F* < 1, η^2^ = 0.003), whereas after induction, there was a significant group difference [*F*_(2,87)_ = 6.003, *p* = 0.004, η^2^ = 0.121]. Specifically, the anger level of the fear group was significantly higher than that of the neutral emotion group (*p* = 0.001) and that of the sad group (*p* = 0.017); however, there was no significant difference between the latter two groups (*p* = 0.360; Figure 3A).

***Positive emotion***

The repeated measures ANOVA of positive emotions found that the main effect of time was significant [*F*_(1,29)_ = 8.183, *p* = 0.005, η^2^ = 0.086; Figure 3C], whereas the main effect of group was not significant (*F* < 1, η^2^ = 0.000). The interaction between time and group was significant [*F*_(2,87)_ = 4.611, *p* = 0.012, η^2^ = 0.096; Figure 3C]. A simple comparison showed that there was no significant difference in positive emotion before and after the sadness induction (*F* < 1, *p* = 0.603). However, the positive emotion level after fear induction was significantly lower than that before fear induction [*F*_(1,29)_ = 3.358, *p* = 0.002], and the positive emotion level after the neutral emotion induction was marginally lower than that before this induction [*F*_(1,29)_ = 1.946, *p* = 0.061].

***Negative emotion***

For the negative emotions indicated by the PANAS, the repeated measures ANOVA found that the main effect of time was not significant [*F*_(1,29)_ = 1.435, *p* = 0.234, η^2^ = 0.016], whereas the main effect of group was significant [*F*_(2,87)_ = 4.955, *p* = 0.009, η^2^ = 0.102]. *Post hoc* multiple comparisons revealed that the negative emotion level of the fear group was significantly higher than that of the neutral emotion group (*p* = 0.002) and marginally higher than that of the sad group (*p* = 0.061). The interaction between time and group was significant [*F*_(2,87)_ = 19.492, *p* = 0.000, η^2^ = 0.309; Figure 3D]. A simple comparison focusing on the group differences showed that before induction, there were no significant group differences in the negative emotion level (*F* < 1, η^2^ = 0.002), whereas after induction, there was a significant group difference in the negative emotion level [*F*_(2,87)_ = 15.774, *p* = 0.000, η^2^ = 0.266]. Specifically, the negative emotion level of the fear group was significantly higher than that of the neutral emotion group (*p* = 0.000) and that of the sad group (*p* = 0.000).

***Aggressive behavior***

Univariate ANOVA of the aggressive behavior showed that the group differences were significant [*F*_(2,87)_ = 4.302, *p* = 0.017, η^2^ = 0.090]. *Post hoc* multiple comparisons of aggression among the three groups are depicted in Figure 3B. The aggression level of the sad group was significantly lower than that of the neutral emotion group (*p* = 0.038) and that of the fear group (*p* = 0.006); however, there was no difference between the neutral group and the fear group (*p* = 0.482).

#### Summary and Discussion of Study 1

In Study 1, we examined whether inducing the emotion of fear and anger could differentially alter (promote or alleviate) the progress of the already evoked anger emotion and related aggressive behavior. Our results partially supported the “fear promotes anger” and “sadness counteracts anger” hypotheses in finding that (a) participants’ subjective angry feeling was greater if they watched fear-inducing movies relative to watching sad or neutral movies and that (b) participants became less aggressive if they watched sad movies relative to watching fear-inducing or neutral movies. One reason why aggressive behavior was not intensified by watching fear-inducing movie clips and the feeling of anger was not reduced by watching sad movie clips could be related to the features of the approach/avoidance motivation contained in the emotion of anger or fear and the MI (rather than the cognitive regulation) nature of sadness to alter the progress of anger. We will discuss this at length in the general discussion.

Relative to the promoting effects of fear on anger, the ameliorating effects of sadness on aggressive behavior may have more practical value because they imply a novel strategy for aggression regulation. However, the AI procedure used by Bushman and colleagues (Bushman et al., 2001; Bushman, 2002), although found to be valid and effective in infuriating participants, might also make participants feel frustrated. These feelings of frustration, according to the Frustration-Aggression Model (Dollard et al., 1939; Geen, 1968; Rule and Percival, 1971; Josephson, 1987), could result in aggressive inclinations, especially when the competitor in the TAP in Study 1 was the person who provided negative comments about the participants’ viewpoint. Therefore, it remains unclear whether sadness can exert general regulatory effects on anger and aggression or can only exert domain-specific regulatory effects on the type of anger and aggressive behavior that has a close relationship with frustration. To investigate this, Study 2 used another anger-inducing procedure, in which participants watched anger-inducing movie clips that were taken from the CEVS database (Xu et al., 2010). This procedure will be largely free of the obvious frustration associated with receiving extremely negative feedback that was present in Study 1.

## Study 2

### Methods

#### Participants

A total of 95 undergraduate and graduate students from universities in Beijing volunteered to participate in this study. The data from five participants were excluded from the final analysis because three participants failed to experience anger and two participants had correctly guessed the purpose of the experiment before it started. The remaining 90 participants (18 males), who ranged in age from 19 to 25 years (with a mean age of 23 years), were all participants who were naive regarding the experimental aims. The participants were randomly assigned to the fear, sadness, and neutral emotion groups, with each group consisting of 30 participants. All of the participants signed the informed consent form for the experiment, and each participant was compensated 15 ∼ 20 RMB for participating in the study.

#### Experimental Design and Procedures

The experimental procedure of Study 2 was the same as that of Study 1 with two exceptions. First, anger was induced by having participants watch anger-inducing movie clips that were taken from the CEVS database by Xu et al. (2010); duration, 2 min 43 s; from the movie “The Tokyo Trial”; intensity, *M* = 2.9362, SD = 1.14) rather than by asking them to read a person’s extremely negative evaluation of their viewpoint. Second, the competitor in the TAP was not a person who had provided negative comments about the participants’ viewpoints; rather, the participants were informed that they would play with a randomly selected person.

### Results

As in Study 1, we performed two repeated measures ANOVA to examine the changes in subjective feelings of anger and positive and negative emotions. We first examined the effects of the AI procedure and then examined the effects of the fear/sadness/neutral emotion induction procedure, all using a 2 (time: second emotional evaluation, third emotional evaluation) × 3 (group: fear group, sad group, neutral emotion group) mixed design. Finally, we performed a one-way ANOVA on competitive game scores to investigate the differences in aggressive behavior among the three experimental conditions.

#### Effects of Anger Induction

***Subjective feelings of anger***

**FIGURE 4:**
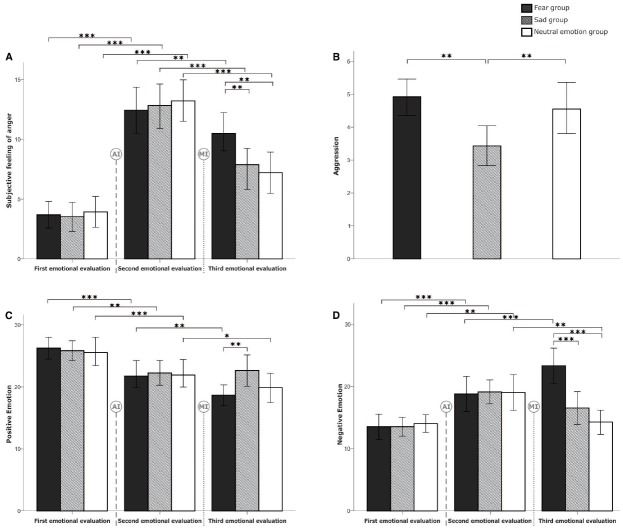
**Comparisons of emotional change and the level of aggressive behavior among the three experimental conditions in Study 2.** Participants’ subjective feelings of anger, positive emotion, and negative emotion in the three experimental stages (i.e., first emotional evaluation, which was administered before anger induction; second emotional evaluation, which was administered after anger induction; and third emotional evaluation, which was administered after the fear/sadness/neutral emotion induction) are shown in Panel **(A,C,D)**, respectively. The levels of aggressive behavior, which were tested after the fear/sadness/neutral emotion induction, are presented in Panel **(B)**. The signs of AI and MI, indicated with a dashed line, represent the anger induction and mood induction manipulations, respectively. The error bars (capped vertical bars) represent 95% confidence intervals. Significant difference at *p < 0.05, **p < 0.01, ***p < 0.001.

The repeated measures ANOVA on feelings of anger showed that the main effect of time was significant [*F*_(1,29)_ = 351.220, *p* = 0.000, η^2^ = 0.801], indicating that the mean level of anger after AI was significantly higher than that before AI. This result confirmed the effectiveness of AI (Figure 4A). However, neither the main effect of group (*F* < 1, η^2^ = 0.006) nor the interaction between time and group (*F* < 1, η^2^ = 0.003) was significant.

***Positive emotion***

The repeated measures ANOVA of positive emotion, as measured by the PANAS positive affect subscale, showed that the main effect of time was significant [*F*_(1,29)_ = 51.125, *p* = 0.000, η^2^ = 0.370; Figure 4C], revealing that the positive emotion level after AI was significantly lower than that before AI. However, neither the main effect of group (*F* < 1, η^2^ = 0.001) nor the interaction between time and group (*F* < 1, η^2^ = 0.005) was significant.

***Negative emotion***

The repeated measures ANOVA of negative emotion, as measured by the PANAS negative affect subscale, showed that the main effect of time was significant [*F*_(1,29)_ = 58.103, *p* = 0.000, η^2^ = 0.400], revealing that negative emotion after AI was significantly increased compared to the level before AI (Figure 4D). However, neither the main effect of group (*F* < 1, η^2^ = 0.002) nor the interaction between time and group (*F* < 1, η^2^ = 0.001) was significant.

In summary, the comparison before and after the induction of anger and the comparison among groups indicated that the manipulation of AI was efficient; it significantly increased the level of anger and negative emotion and decreased the level of positive emotion. Furthermore, there was no significant group difference in feelings of anger, positive emotion, and negative emotion before or after the AI.

#### Effects of Fear and Sadness Induction on Anger and Aggressive Behavior

***Subjective feelings of anger***

The repeated measures ANOVA of anger level revealed a significant main effect of time [*F*_(1,29)_ = 92.132, *p* = 0.000, η^2^ = 0.514]. The anger level after these fear/sadness/neutral emotion inductions was significantly lower than that before the emotion inductions. The main effect of group was not significant [*F*_(2,87)_ = 1.255, *p* = 0.290, η^2^ = 0.028]. The interaction between time and group was significant [*F*_(2,87)_ = 7.886, *p* = 0.001, η^2^ = 0.153]. A simple comparison that focused on the group differences showed that before induction, there was no significant group difference (*F* < 1, η^2^ = 0.006), whereas after induction, there was a significant group difference [*F*_(2,87)_ = 6.334, *p* = 0.003, η^2^ = 0.127]. Specifically, the anger level of the fear group was significantly higher than that of the neutral emotion group (*p* = 0.001) and that of the sad group (*p* = 0.006); we observed no differences between the latter two groups (*p* = 0.622; Figure 4A).

***Positive emotion***

The repeated measures ANOVA of positive emotion indicated that the main effect of time was significant [*F*_(1,29)_ = 12.322, *p* = 0.001, η^2^ = 0.124; Figure 4C], whereas the main effect of group was not [*F*_(2,87)_ = 1.302, *p* = 0.277, η^2^ = 0.029]. The interaction between time and group was significant [*F*_(2,87)_ = 4.745, *p* = 0.011, η^2^ = 0.098; Figure 4C]. A simple comparison of the group differences showed that before induction, there was no significant group difference (*F* < 1, η^2^ = 0.0006), whereas after induction, there was a significant group difference [*F*_(2,87)_ = 3.491, *p* = 0.035, η^2^ = 0.074]. Specifically, the positive level of the sad group was marginally significantly higher than that of the neutral emotion group (*p* = 0.074) and significantly higher than that of the fear group (*p* = 0.012); however, there was no difference between the latter two groups.

***Negative emotion***

The repeated measures ANOVA of negative emotion revealed that the main effect of time was not significant (*F* < 1, η^2^ = 0.007) but that the main effect of group was significant [*F*_(2,87)_ = 5.415, *p* = 0.006, η^2^ = 0.111]. *Post hoc* multiple comparisons revealed that the negative emotion level of the fear group was significantly higher than that of the neutral emotion group (*p* = 0.002) and that of the sad group (*p* = 0.031). The interaction between time and group was significant [*F*_(2,87)_ = 16.662, *p* = 0.000, η^2^ = 0.227; Figure 4D]. A simple comparison of the group differences showed that before induction, there was no significant group difference in the negative emotion level [*F* < 1, η^2^ = 0.001], whereas after induction, there was a significant group difference in the negative emotion level [*F*_(2,87)_ = 15.604, *p* = 0.000, η^2^ = 0.264]. Specifically, the negative emotion level of the fear group was significantly higher than that of the neutral emotion group (*p* = 0.000) and that of the sad group (*p* = 0.000).

***Aggressive behavior***

Univariate ANOVA of aggressive behavior showed that the group differences were significant [*F*_(2,87)_ = 6.169, *p* = 0.003, η^2^ = 0.124]. *Post hoc* multiple comparisons of aggression among the three groups are depicted in Figure 4B. The aggression level of the sad group was significantly lower than that of the neutral emotion group (*p* = 0.007) and that of the fear group (*p* = 0.002); there was no difference between the neutral group and fear group.

#### Summary and Discussion of Study 2

Study 2 replicated the main findings of Study 1, indicating the promoting effects of the emotion of fear on angry feelings and the alleviating effects of the emotion of sadness on aggressive behavior. Thus, Study 2 demonstrated that the experimental phenomenon that was observed in Study 1 can also be observed when anger is induced without feelings of frustration and when the competitor in the game that is used to test aggression is not a person who gave the participants very negative feedback. However, in contrast to Study 1, Study 2 found that subjects’ positive affect after watching sad movie clips was significantly or marginally higher than that after watching fear-inducing or neutral emotion movie clips. This difference could be related to the method of AI, which had significantly reduced positive affect. In Study 1, participants read another person’s very negative comments on their viewpoints, whereas in Study 2, they watched anger-inducing movie clips. The decrease in positive affect that was produced by watching anger-inducing movie clips could be relatively easily altered by watching sad movie clips, whereas the decrease in positive affect produced by reading very negative feedback of one’s own viewpoint is relatively difficult to alter because this decrease was caused by negative feedback that might directly affect one’s self-efficiency. The results of Study 2 implied that the emotion of sadness could help one recover his/her positive affect that was reduced by the emotion of anger, at least when this emotion was not directly related to one’s self-efficiency. The promoting effects of the emotion of sadness on positive affect were also consistent with previous studies, which found that the neural basis of sadness was similar to that of happiness (Panksepp, 1998; Wager et al., 2015).

## General Discussion

In these two experiments, we made two key experimental observations that were mostly consistent with the view of traditional Chinese philosophy and medicine of a mutual promotion and counteraction relationship among the different types of emotions. First, the angry feeling in the fear group was significantly higher than that in the sad and neutral emotion groups, which supported the hypothesis of “fear induces anger.” Second, the aggressive behavior in the sad group was significantly lower than that in the other two groups, supporting the hypothesis that “sadness overcomes anger.” Given that the angry feeling in the three groups was all reduced after watching the movie clips, the MI effects that we observed in this study could be related to a distraction effect, which refers to when individuals attempt to focus their attention on a specific topic or task such that attention is shifted away from the original topic or task, resulting in the reduction of negative emotions (Gross and Kalra, 2002). However, the distraction perspective could not account for the group differences between the three conditions because the distraction that the three groups experienced was nearly comparable in their duration and task feature (i.e., passively viewing short movie clips). An essential factor that should be considered, however, is how the induced emotion (i.e., fear or sadness) interacts with the to-be-regulated emotion (i.e., anger).

First, the interaction between anger and fear might promote anger given that both types of emotion were induced by the perception of threat; thus, the induction of fear over the already evoked emotion of anger could increase one’s feelings of anger. A recent meta-analysis on brain activation patterns of different emotion categories found that the cortical and amygdala patterns involved in fear are similar to those involved in anger (Wager et al., 2015). Taking this finding one step further, anger and fear categories both preferentially engage cortical processes that support an “external orientation/object-focused” schema that is characterized by goal-driven responses for which objects and events in the world are in the foreground (Wager et al., 2015). However, fear can also differ from anger in terms of the inclination to escape rather than to approach (Izard et al., 1984). Given that the approach motivation was important in inspiring the angered individuals to become aggressive (Harmon-Jones and Peterson, 2008), it was natural to find that the induction of fear did not significantly increase the level of aggressive behavior but did increase the level of anger. Although the angry feeling after the induction of fear was significantly higher than that after the induction of sadness or neutral emotion, the angry feeling in all three groups was reduced after watching the movie clips. The fact that the induction of fear did not cause the provoked individual to become angrier than they previously were implied that the promoting effect of fear on anger was not sufficiently robust to override the natural decline of the first AI; however, it did slow the recovery from the AI.

Second, the interaction between anger and sadness might alleviate anger or the related aggressive behavior. This is because the cognitive and emotional elements of sadness and anger were so dissimilar that the activation remaining in the anger- or aggression-related circuits could be more efficiently erased by the activation associated with sadness. In support of this possibility, a meta-analysis showed that in anger, the frontoparietal cortex is co-activated positively with the amygdala and cerebellum and the dorsal attention network is negatively associated with cerebellar activation; however, sadness is characterized by a lack of co-activation between cortical and subcortical cerebellar/brainstem networks and a strong, preserved co-activation of hindbrain (cerebellar/brainstem) systems (Wager et al., 2015). Moreover, in contrast to anger, sadness was found to engage cortical patterns that support an internal orientation/homeostatic-focused schema that was characterized by orientation to immediate somatic or visceral experience, which prioritizes the processing of interoceptive and homeostatic events (Wager et al., 2015). Notably, although the aggressive behavior of the sad group was significantly lower than that of the neutral group, angry feelings did not show such a difference. The reason that sadness failed to reduce people’s angry feelings could be related to the feature of this distraction regulatory strategy. Different from the top-down regulatory strategy, such as cognitive reappraisal, which directly changes people’s cognition of the unpleasant event (Ochsner et al., 2002), the induction of sadness did not alter the participants’ attitude toward the anger-inducing event that they encountered earlier. In other words, the participants in the sad group were likely to ruminate on the “inexplicable bullied incident” and remain in the angry state, although their internal impulse to punish their partner (i.e., the aggressive behavior) had indeed decreased (relative to the neutral group).

## Conclusion

In summary, although previous studies have indicated that different types of emotion (such as anger and sadness) can alter people’s cognitive style, such as that observed in stereotypic judgments and causal judgments (Keltner et al., 1993), no direct evidence has been provided to show that different types of emotion can have different regulatory effects on a target emotional state. The present study was the first to demonstrate that angered participants show a lower level of aggressive behavior if the emotion of sadness is later induced and that they show a higher level of anger if the emotion of fear is induced. These outcomes not only provide evidence for the mutual promoting and counteracting hypotheses of emotions but also imply that a new emotion regulation approach can be developed to modulate unwanted emotions using a “cognition free” strategy.

### Conflict of Interest Statement

The authors declare that the research was conducted in the absence of any commercial or financial relationships that could be construed as a potential conflict of interest.
